# Intra-voxel angular dispersion of fibers in corpus callosum decreases with healthy aging

**DOI:** 10.1162/imag_a_00463

**Published:** 2025-01-30

**Authors:** Hunter G. Moss, Andrew A. Chen, Jens H. Jensen, Andreana Benitez

**Affiliations:** Center for Biomedical Imaging at the Medical University of South Carolina, Charleston, SC, United States; Department of Neuroscience at the Medical University of South Carolina, Charleston, SC, United States; Department of Public Health Sciences at the Medical University of South Carolina, Charleston, SC, United States; Department of Radiology at the Medical University of South Carolina, Charleston, SC, United States; Department of Neurology at the Medical University of South Carolina, Charleston, SC, United States

**Keywords:** axon, corpus callosum, fiber orientation density function, aging, fiber ball imaging, diffusion MRI

## Abstract

The goal of this study was to investigate how the angular dispersion of axonal fibers changes during the course of healthy aging. The angular dispersion was derived from the fiber orientation density function (fODF), which was estimated in vivo with a diffusion MRI technique called fiber ball imaging. Intra-voxel angular dispersion of axonal fibers within the corpus callosum at the midline up to the anterior tip of the frontal horn of the lateral ventricles was quantified for a cohort of 63 healthy older adults (ages 45 to 85 years). The splenium, body, and genu of the corpus callosum were examined separately, and fODFs within each of these regions were averaged across voxels to obtain three mean fODFs for each study participant. For all three regions, we found that the angular dispersion, as quantified by the full width of the mean fODF at half its maximum, decreases significantly with age. However, these decreases were not significantly different across the regions. In addition, the heights of the mean fODF peaks increase with age. This reduction in angular dispersion and increase in height imply axons with orientations deviating further from the fODF peak are more likely to be lost in the course of healthy aging. We propose that this is related to the known preferential loss of thinner myelinated axons with increasing age.

## 1 Introduction

Even in healthy aging, cerebral white matter undergoes striking changes, including reduction in volume, axonal degeneration, and development of white matter hyperintensities (Blinkouskaya et al., 2021;Liu et al., 2017;Tang et al., 1997). Characterization of these changes can provide a normative reference to help understand the progression of neuropathologies. For example, an association between Alzheimer’s disease and reduced volume of several white matter regions has been found by comparison with healthy aging data (Salat et al., 2009). In addition, age-related white matter changes may be clinical risk factors for dementia and other neurological disorders (Bahrani et al., 2017;Mortamais et al., 2013).

A notable aspect of age-related axonal degeneration is that the number of myelinated axons with diameters greater than about 1.5 μm is largely preserved even as the number of thinner axons is sharply reduced (Marner et al., 2003). As a consequence, the average diameter of axons increases significantly with age, as has been verified specifically for the corpus callosum (CC) using both histology (Aboitiz et al., 1996) and diffusion MRI (dMRI) (Fan et al., 2019). This change in the distribution of axon diameters strongly suggests that aging may also affect other population characteristics of axon microstructure. One such population characteristic that can also be measured with dMRI is the intra-voxel distribution of axon orientations, as is typically quantified in terms of the fiber orientation density function (fODF) (Dell’Acqua & Tournier, 2019;Moss & Jensen, 2022a;Tournier et al., 2004). The purpose of this paper is to investigate how fODFs evolve during the course of healthy aging. Since fODFs estimated from dMRI are commonly used as building blocks for constructing white matter fiber tractography (Lazar, 2010;Tournier, 2019), altered fODFs imply changes in the brain’s structural connectivity and thus may be relevant to age-related cognitive decline (Fjell et al., 2017;Li et al., 2020).

In this study, we only examine fODFs within the midline portion of the CC in order to avoid complexities associated with intersecting fiber bundles, as are found in many other white matter regions (Jeurissen et al., 2013). At the midline CC, fODFs primarily reflect angular dispersion of the axonal fibers within an imaging voxel relative to a single main direction. For selected parts of the CC, the study ofRonen et al. (2014)finds the standard deviation of the dispersion to be 18.1° ± 4.6° based on histology and 18.6° ± 3.0° from diffusion magnetic resonance spectroscopy of N-acetylaspartate. This is comparable with the angular resolution achievable with high-fidelity fODFs estimated from dMRI using strong diffusion weightings (Moss & Jensen, 2022a). Therefore, changes in angular dispersion of several degrees or more should be detectable with this approach.

Of the various dMRI methods available for determining fODFs, here we employ one referred to as fiber ball imaging (FBI) (Jensen et al., 2016;Moss et al., 2019;Moss & Jensen, 2022a). To estimate the fODF, FBI applies the inverse Funk transform, a linear operation, directly to the dMRI data in each voxel. The validity of FBI relies on the 1/$\sqrt{b}$power-law scaling of the dMRI signal in the intra-axonal space at large*b*-values (*b*≥ 4000 s/mm^2^) (McKinnon et al., 2017;Moss et al., 2019;Veraart et al., 2019). Conventional dMRI techniques (e.g., diffusion tensor imaging, DTI (Basser et al., 1994); diffusional kurtosis imaging, DKI(Jensen et al., 2005)) are distinguished from FBI in not incorporating biophysical modeling assumptions. Therefore, commonly used diffusion measures, such as the fractional anisotropy (FA), do not have explicit connections to tissue properties, including the fODF.

Among the more advanced dMRI analysis methods, q-ball imaging (Tuch, 2004) is most closely related to FBI. However, q-ball imaging estimates a diffusion ODF, reflecting the directional dependence of water diffusion, rather than an fODF. Compared with alternative fODF estimation methods such as constrained spherical deconvolution (CSD) (Tournier et al., 2007) or neurite orientation dispersion and density imaging (NODDI) (Zhang et al., 2012), the advantage of FBI is its simplicity. For instance, CSD requires an empirically determined response function and numerical regularization, while NODDI makes a priori assumptions about the fODF shape along with nonlinear fitting to a model. Although requiring a larger*b*-value than DTI, DKI, CSD, or NODDI, FBI provides robust fODF estimates that are based on minimal assumptions and involve only straightforward mathematical procedures.

Microstructural models, such as neurite orientation dispersion and density imaging (NODDI) (Zhang et al., 2012), have been applied in healthy aging to investigate axon dispersion using the orientation dispersion index (ODI). Prior studies have reported that ODI correlates with age throughout the white matter (Bauer et al., 2022;Cox et al., 2016). However, in the CC specifically, correlation results have been directionally inconsistent. For instance, positive correlations of ODI with age were reported byBilliet et al. (2015)andMotovylyak et al. (2022), while negative correlations with age were found byRaghavan et al. (2021). These inconsistencies may stem from the underlying framework used in many microstructural models consisting of arbitrary initializations, nonlinear fitting routines, and fODF shape constraints. We improve upon prior work to study dispersion changes with age by using FBI, which does not require such a model framework as previously explained.

In this study, we apply FBI to a cohort of 63 healthy older adults with ages ranging from 45 to 85 years whose data were published as part of a prior study (Dhiman et al., 2022). Compared with prior work, our study cohort is unique in that it excludes incipient disease factors that may confound the analysis. To allow for direct comparison across voxels and participants, the fODFs are rotated from the laboratory coordinates to a local frame of reference determined by each fODF’s particular structural features (Moss & Jensen, 2022a). For each participant, the fODFs for voxels from the splenium, body, and genu of the CC are then averaged separately. The three mean fODFs are correlated with age across participants to estimate the extent to which angular dispersion changes with age. These associations are also quantified with a novel linear regression analysis that gives the rate of change as a function of direction. We consider the splenium, body, and genu separately since prior histological (Aboitiz et al., 1996) and imaging (Dhiman et al., 2022;Fan et al., 2019) studies have revealed differences in the impact of aging on their microstructure.

## 2 Methods

### 2.1 Participants

This study employs baseline data from a subsample of community-dwelling older adults (N = 63; 71.4% female; average age: 64.5 ± 8.6 years; age range: 45.1 – 84.7 years) gathered as part of an ongoing longitudinal study of preclinical Alzheimer’s disease and published in a prior study on dMRI white matter changes in healthy aging (Dhiman et al., 2022). Briefly, in this study, healthy aging is defined as (1) a negative florbetapir PET amyloid scan as determined by a certified reading radiologist, (2) a mean bi-hemispheric medial temporal atrophy score less than 2 (DeCarli et al., 2007;Scheltens et al., 1992), and (3) a Fazekas scale score less than 2, indicating non-significant hyperintensities in periventricular or deep white matter (Fazekas et al., 1987), with board-certified neuroradiologists providing all ratings using T1 and T2-FLAIR MRI. Furthermore, all participants have intact cognition defined as a Montreal Cognitive Assessment (MoCA) (Nasreddine et al., 2005) score greater than or equal to 23 (Carson et al., 2018;Luis et al., 2009). All participants have provided written informed consent for the study, which is approved by the institutional review board at the Medical University of South Carolina.

### 2.2 Image acquisition

Axial brain images were acquired on a 3T Prisma^fit^scanner (Siemens Healthineers, Erlangen, Germany) using 32-channel head coil and a single-shot twice refocused echo-planar imaging dMRI sequence (Reese et al., 2003). Data were gathered at*b*-values of 1000 and 2000 s/mm^2^with 30 diffusion-encoding directions for a DKI analysis (Jensen & Helpern, 2010). FBI data were gathered at a*b*-value of 6000 s/mm^2^with 128 diffusion-encoding directions. The following imaging parameters were matched across the DKI and FBI protocols: 3 mm isotropic voxels, TE/TR = 95/4800 ms, 74 × 74 acquisition matrix, 42 axial slices, bandwidth of 1648 Hz/px, and slice acceleration/parallel imaging factors = 2/2 with anterior-to-posterior phase encoding. An additional 22*b*= 0 s/mm^2^images were obtained with these same settings, and a single reversed phase encoding*b*= 0 image was acquired for distortion correction.

### 2.3 Image processing

All dMRI data were processed using PyDesigner (Dhiman et al., 2021,^2024^), an open-source Python-based software based on the DESIGNER pipeline that performs preprocessing steps to improve accuracy and precision, including denoising, Gibbs ringing artifact correction, distortion correction, motion correction, CSF-excluded NaN-smoothing with a Gaussian kernel of 1.25 × (voxel size), and Rician noise bias correction (Ades-Aron et al., 2018). PyDesigner also generated parametric maps of mean diffusivity (MD) and mean kurtosis (MK) from the DKI data and fODFs from the FBI data with the diffusivity scale set to$D_{0}=3$µm^2^/ms (Holz et al., 2000). The mean signal-to-noise ratio (SNR) across all subjects in white matter was 48 ± 9, 27 ± 4, 14 ± 3, and 6 ± 1 for*b*-values of 0, 1000, 2000, and 6000 s/mm^2^, respectively. Coregistration was done between the DKI and FBI data sets since they were gathered sequentially but separately. The scans took 11 min 26 s (FBI) and 5 min 51 s (DKI) each, during which unconscious head movement is possible.

The fODFs were represented as spherical harmonic expansions that included all even degree terms up to and including degree 8; odd degree terms vanish due to antipodal symmetry (Jensen et al., 2016;Moss et al., 2019;Moss & Jensen, 2022a). The maximum degree for the expansion was chosen based on a prior study (Moss & Jensen, 2022b), which showed a reduction in fODF aliasing errors when the number of diffusion encoding directions is two to three times oversampled compared with the number of harmonic coefficients. Oversampling is especially important for FBI since errors propagate forward to the fODF via the inverse Funk transform which amplifies higher degree harmonics. Having 128 encoding directions gives us an oversampling factor of 2.8 for degree 8 containing 45 harmonic coefficients. The choice of maximum degree also sets the resolving power which results in an angular resolution of about 28° for the diffusivity scale used here (Moss & Jensen, 2022a). The angular resolution is defined as the observed full-width-at-half-maximum (FWHM) for an fODF with no angular dispersion. Typically, the FWHM is expected to be larger than this for an fODF peak with a nonzero dispersion. If additional harmonic degrees were included, the angular resolution could be reduced, but the fODFs would then be more prone to noise and aliasing artifacts.

The DKI and FBI maps from each participant were registered into a common space by using the method ofRaffelt et al. (2011)as implemented by MRTrix3 (Tournier et al., 2019). This registration algorithm takes advantage of the structural information contained in the fODFs to improve white matter alignment. As recommended, the transformation to the common space was determined using the fODFs’ spherical harmonic coefficients only up to degree 4, even though the full fODFs were registered. Furthermore, the voxel size of the template was kept identical to the original data in order to keep from introducing any interpolation artifacts unnecessarily into the fODFs during template creation (e.g., registering into anatomical T1-space).

After registration, all fODFs were rotated into a local frame of reference defined by their individual structure (Moss & Jensen, 2022a) and then optimally rectified to eliminate nonphysical negative values (Moss & Jensen, 2021a). In the local frame, fODFs are consistently aligned to allow for direct quantitative comparison across voxels and participants. All fODFs are normalized so that their integral over all directions is unity. Thus, they may be interpreted as probability density functions for the axon orientations. Although not firmly established, it is thought that fODFs estimated with FBI reflect primarily myelinated axons (Moss et al., 2019), which predominate in the CC (Lamantia & Rakic, 1990).

### 2.4 Visualization of fODFs

Since fODFs are defined as functions on a spherical surface, hemispheric equidistant azimuthal projection (HEAP) maps were used for two-dimensional graphical display of fODFs (Moss & Jensen, 2022a), with this specific projection being chosen to minimize geometric distortion (Gott et al., 2021). Because of antipodal symmetry, HEAP maps represent the full fODF structure, even though they only show a single hemisphere. The maps appear as circular disks, with the radial distance from the center equal to the polar angleθfor spherical coordinates defined in the three-dimensional local frame and with the two-dimensional polar angle equal to the azimuthal angleϕof the spherical coordinates. Thus, the disks have a radius ofπ/​2=90°, and the line defined bysinϕ=0corresponds to the*x*-axis of a HEAP map while the linecosϕ=0corresponds to the*y*-axis. The HEAP map coordinate system is illustrated byFigure 1. In this paper, the terms “along the*x*-axis” and “along the*y*-axis” are in reference to this two-dimensional representation.

**Fig. 1.**
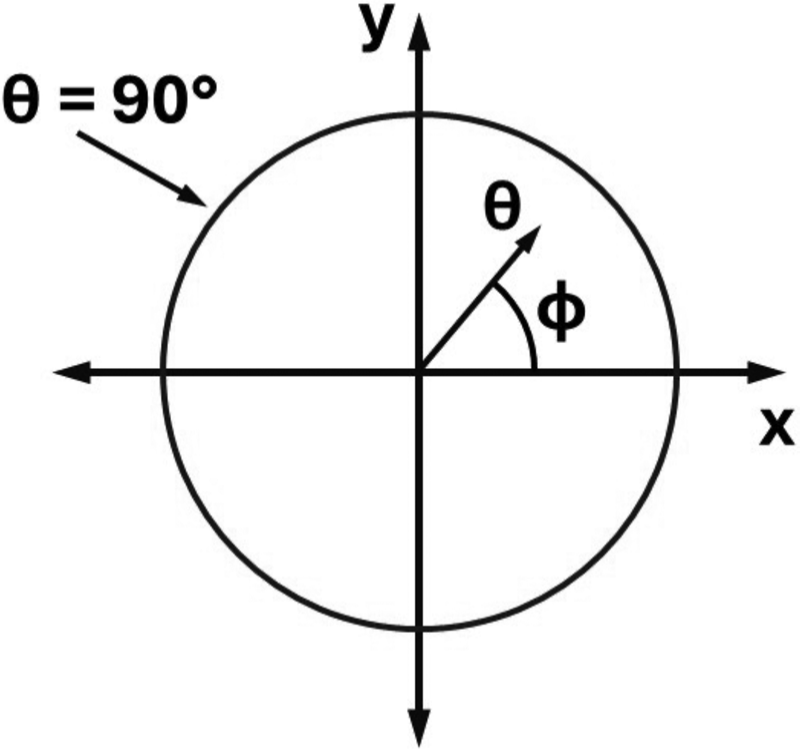
Coordinate system for HEAP map representation of fODFs. By definition, fODFs are functions on a spherical surface, and they are typically described in terms of spherical coordinates. For plotting purposes, HEAP maps can be used to depict fODFs in two dimensions with minimal geometric distortion. In this projection, the fODFs appear as circular disks. The distance from the center of the disk is set equal to the polar angleθof the spherical coordinate system, and the polar angle of the HEAP map relative to the*x*-axis is set equal to the azimuthal angleϕof the spherical coordinate system. Since fODFs inherently possess antipodal symmetry, a single hemisphere is sufficient to fully specify any fODF. The edge of the disk corresponds toθ=90°, the*x*-axis corresponds to the linesinϕ=0, and the*y*-axis corresponds to the linecosϕ=0.

### 2.5 Regions-of-interest analysis

Study-specific MD and MK templates were created by averaging across participants in the common space. A binary white matter mask was then defined as all voxels inside the cerebrum with MK > 0.9 and MD < 1.5 μm^2^/ms. The MK threshold is similar to that of prior work (Yang et al., 2013) but reduced slightly in order to accommodate the observed decrease in MK with age (Dhiman et al., 2022). The MD threshold is applied to minimize partial volume contamination from CSF. The Johns Hopkins’ ICBM-DTI-81 white matter atlas (Mori et al., 2005) was then transformed into the common space by using an affine transform with FSL and nearest neighbor interpolation (Jenkinson et al., 2002,^2012^). Three structures within the CC—the splenium, body, and genu—were then selected from the transformed Johns Hopkins white matter atlas and a region of interest (ROI) for each structure was defined as all voxels contained within both the structure and the white matter mask. These ROIs are illustrated inFigure 2.

**Fig. 2.**
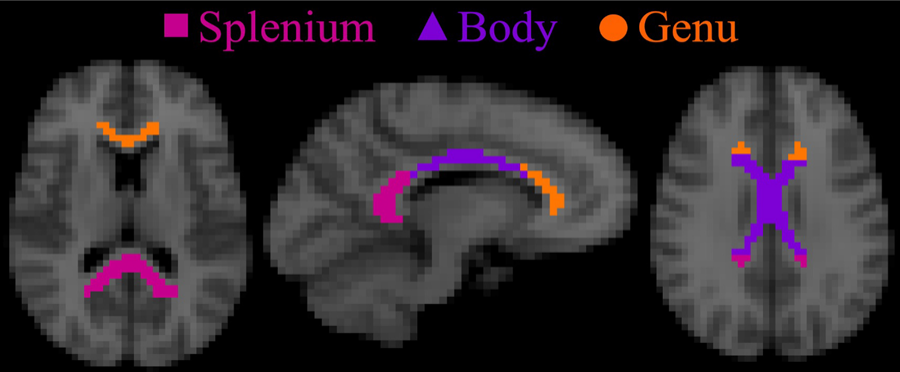
The three ROIs within the midline CC used in this study are depicted in the common space. The splenium (magenta), body (purple), and genu (orange) are defined by using a standard brain atlas together with a white matter mask based on the MD and MK maps. The application of the white matter mask helps to reduce partial volume effects from gray matter and cerebrospinal fluid.

### 2.6 Statistical analysis

To quantify angular dispersion, we define FWHM*_x_*as the FWHM of an fODF along the*x*-axis and FWHM*_y_*as the FWHM of an fODF along the*y*-axis. We also define the center peak height,$F_{0}$, as the value of an fODF at the pointθ=0, which corresponds to the center of the circle shown inFigure 1. First, for each participant, we calculated*F*_0_, FWHM_*x*_, and FWHM_*y*_from the fODF of each voxel in the CC ROI. To compare with prior investigations of intra-voxel dispersion (Mollink et al., 2017), we performed a voxel-wise average across the participants for each metric. Next, to assess the age dependence of the fODFs, mean fODFs for each participant were obtained by averaging the fODFs over all the voxels within each of the three CC ROIs. Pearson’s correlation coefficient (*r*) was used to assess how these three quantities depend on age for the participants’ mean fODFs within each of the three ROIs. Statistical significance was determined using the Benjamini–Hochberg procedure (Benjamini & Hochberg, 1995) for nine comparisons with a false discovery rate of 0.05.

To further quantify the age dependence of the fODFs, we analyzed the spherical harmonic coefficients individually since they are fundamental to the overall structure of the fODF. The harmonic coefficients in the local reference frame can be considered rotational invariants as they are independent of the laboratory coordinates. To do this, the spherical harmonic coefficients (in their real form) of the fODFs from the individual participants were separately correlated with age for degree (l) and order (m) in each of the three ROIs. This included degreesl=2,4,6,8and ordersm=−l,−l+1,…,l. The odd degree coefficients were not considered as these all vanish by symmetry, and the coefficient forl=0andm=0has no age dependence as it merely reflects the fODF normalization. Thus, considering the fact that in the local frame of reference the coefficients for*l*= 2 and*m*= -2, -1, and 1 also vanish, 41 spherical harmonic coefficient Pearson’s correlations were calculated for each ROI. Statistical significance across these tests was determined intra-regionally using the Benjamini–Hochberg procedure with a false discovery rate of 0.05.

We use direction-specific linear regressions to model the mean fODFs within each ROI across age. To describe this, we denote the mean fODF for participant*i*by$F_{i}(\text{u})$, where the index*i*runs from 1 to*N*, with*N*being the total number of participants, and**u**is the direction in the local frame given by the spherical angles (θ,ϕ). Additionally, let$t_{i}$be the age of participant*i*minus the average age (64.5 years) for the full group of participants. The linear model for the fODF with respect to age can then be expressed as

$$F_{i}(\text{u})=\bar{F}(\text{u})+t_{i}G(\text{u})+\#(\text{u}),$$ (1)

where$\bar{F}(\text{u})$is the group-averaged mean fODF acting as a direction-specific intercept,G(u)is the direction-specific rate of change in the fODF across age, and#(u)are errors assumed to be normally distributed. For this model, we estimated, in each direction, the rate of change and Pearson’s correlation coefficient. FromEquation 1, we calculated the predicted fODFs at ages 45, 65, and 85 years. Additionally, the angular dispersions FWHM*_x_*and FWHM*_y_*for the predicted fODFs were calculated as a function of age.

## 3 Results

The voxel-wise group-average*F*_0_, FWHM_*x*_, and FWHM_*y*_are presented inFigure 3for identical image slices as inFigure 2. The largest values of*F*_0_are located mainly in the core region of each ROI, except for a noticeable dip in voxels of the body along the interhemispheric fissure line. The lowest*F*_0_values are seen in voxels of the anterior genu, posterior splenium, and superior body (top row). On average, values for*F*_0_(avg. ± SEM)—genu (0.78 ± 0.28), body (0.79 ± 0.33), and splenium (0.76 ± 0.22)—are consistent across the regions. For the angular dispersion as quantified by the FWHM_*x*_(middle row) and FWHM_*y*_(bottom row), a low-high-low pattern is observed in the lateral-medial direction of the body (right panels). This pattern is more pronounced in the FWHM_*x*_. In contrast, the angular dispersion throughout the splenium and genu is relatively stable with high FWHM_*x*_and low FWHM_*y*_. However, the regional averages for FWHM_x_and FWHM_y_—genu (38° ± 11° and 31° ± 7°), body (34° ± 10° and 31° ± 9°), and splenium (40° ± 10° and 32° ± 7°)—are again relatively consistent, though on average the body has lower FWHM_*x*_.

**Fig. 3.**
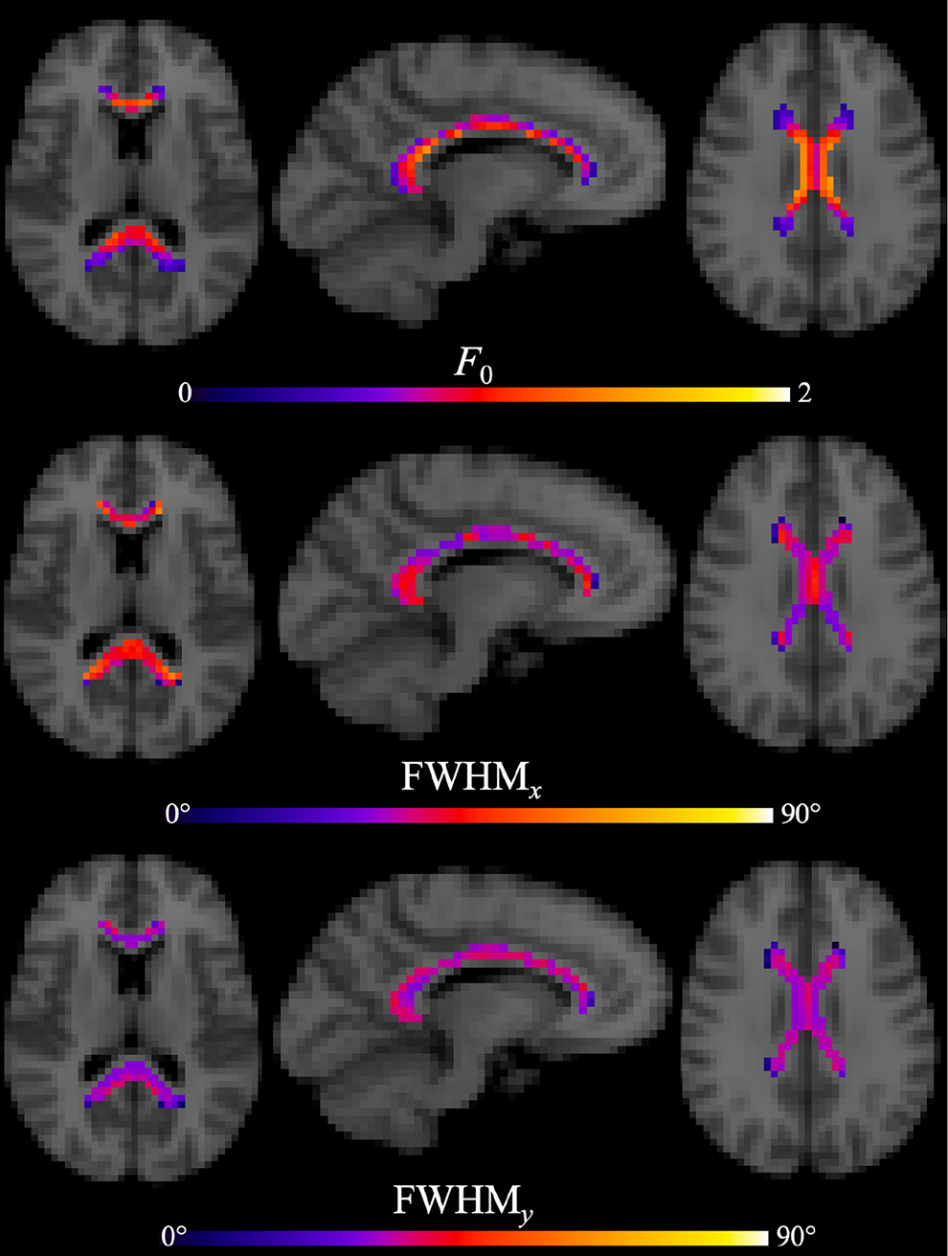
Voxel-wise group averages within the entire CC for the fODF peak amplitude (*F*_0_) and intra-voxel angular dispersion (FWHM_*x*_and FWHM_*y*_) are displayed. Note the image slices shown are identical to those inFigure 2. The largest values of*F*_0_are located mainly in the central region of each ROI, except for the voxels of the body along the interhemispheric fissure line, which show a slight decrease in*F*_0_. In contrast, the lowest values are observed to be on the periphery for the genu and splenium. The values for FWHM_*x*_and FWHM_*y*_show a distinct low-high-low pattern in the body but are relatively stable throughout the genu and splenium. The low-high-low dispersion pattern seen in the body is qualitatively similar to that reported byMollink et al. (2017).

Examples of mean fODFs for the splenium, body, and genu from three selected participants are shown inFigure 4. Note that the fODFs from the oldest participant (84.7 years) are distinctly sharper than for the youngest participant (45.4 years). This sharpness is a consequence of both a decrease in FWHM_*x*_and an increase in*F*_0_. The fODFs are more elongated along the*x*-axis (horizontal direction) than along the*y*-axis (vertical direction) as a consequence of being rotated into the local frame of reference. Note that the visible contour lines here and elsewhere are only for visualization purposes and are not to be interpreted quantitatively.

**Fig. 4.**
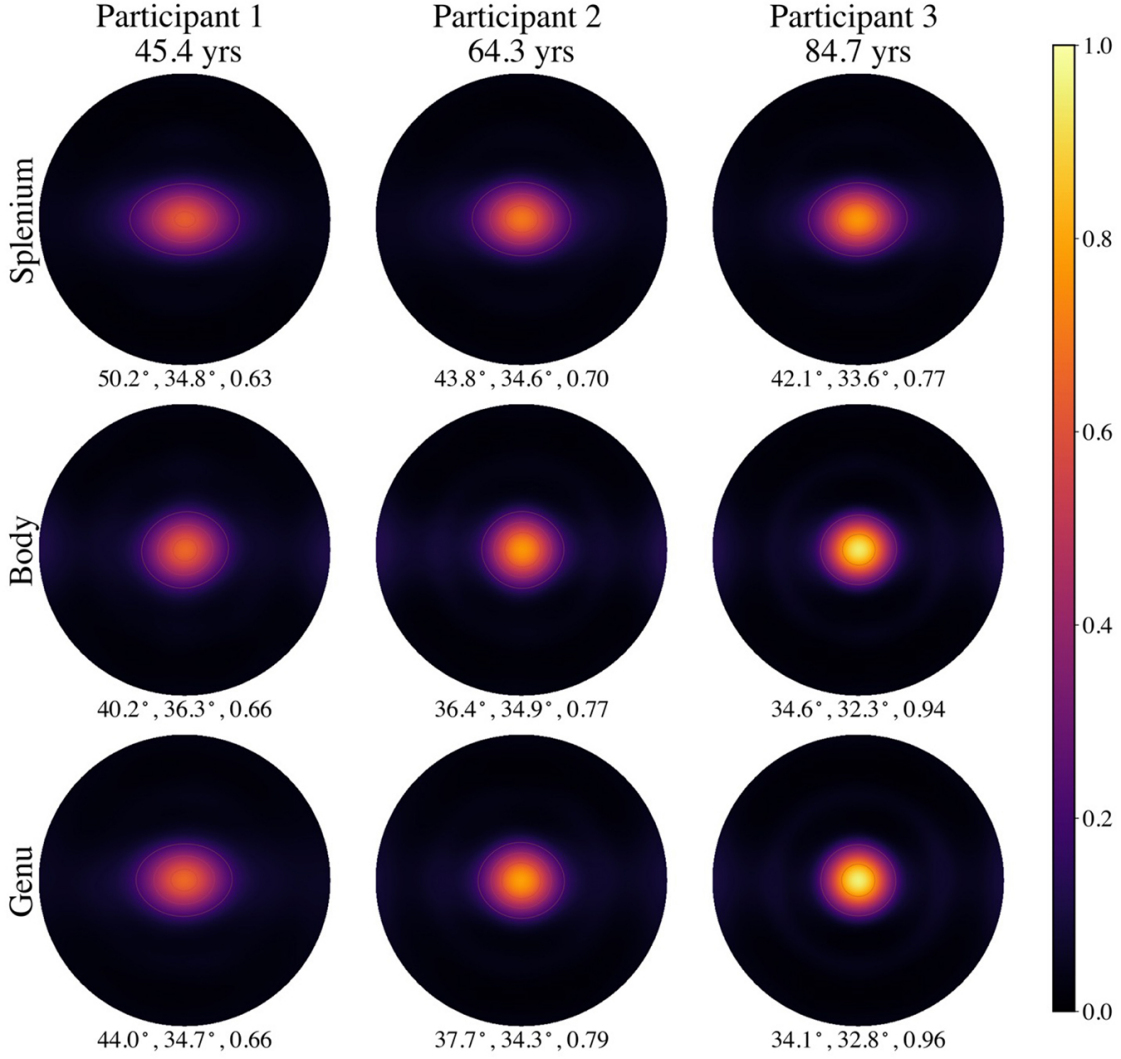
Examples of mean fODFs from three individuals of different ages displayed in the local frame of reference as HEAP maps. The three numbers listed below each HEAP map indicate, respectively, the angular dispersion along the*x*-axis (FWHM*_x_*), angular dispersion along the*y*-axis (FWHM*_y_*), and the center peak height (*F*_0_). In the local frame of reference, FWHM*_x_*is greater than FWHM*_y_*. All fODFs are normalized so that their integral over all directions is unity. The visible contour lines on the HEAP maps indicate amplitude bands and are only for visualization purposes.

**Fig. 5.**
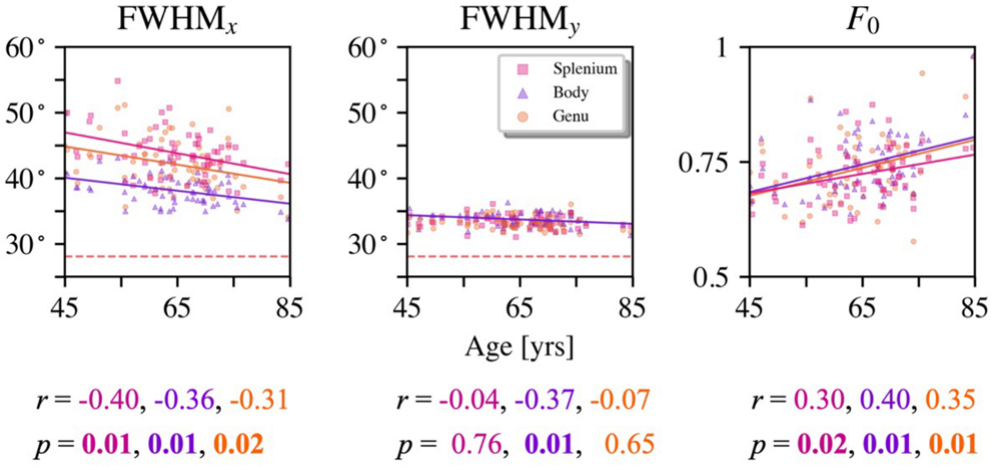
The fODF summary statistics of FWHM*_x_*, FWHM*_y_*, and$F_{0}$from three regions of the CC for all 63 subjects. FWHM*_x_*decreases substantially with age while the dispersion along the*y*-axis shows a more modest age-related reduction. The center peak height increases significantly with age in all three regions. The Pearson’s correlation coefficients (r) that are significant after correction for multiple comparisons have bolded*p*-values. This represents all cases except for FWHM*_y_*in the genu and splenium. For the statistics showing significant correlations, the linear least squares best fits are indicated with solid lines. The dashed horizontal lines show the angular resolution of the fODF estimation for a maximum spherical harmonic degree of 8, as used in this analysis.

Figure 5plots the angular dispersions as a function of age in the three ROIs for all 63 participants as quantified by FWHM*_x_*(left panel) and FWHM*_y_*(center panel). Also shown are the center peak heights (right panel). The correlation coefficient varies from -0.32 to -0.40 for FWMH*_x_*, from -0.08 to -0.28 for FWHM*_y_*, and from 0.31 to 0.40 for$F_{0}$, reflecting a decrease in dispersion with age and an increase in peak height. After correction for multiple comparisons, all of these correlations are significant except for FWHM*_y_*in the genu and splenium. The solid lines are linear least squares best fits for data having a significant correlation. The horizontal dashed lines in the left and center panels indicate the angular resolution corresponding to our choice of maximum degree for the spherical harmonic expansion, which is about 28°. All measured angular dispersions obtained using our experimental and analysis procedures are expected to be larger than the angular resolution even if the true angular dispersion was smaller.

The Pearson correlations for the spherical harmonic coefficients with age are shown inFigure 6, where the numbers indicate the magnitude and the colors indicate the sign (red = positive, blue = negative). Significant correlations are indicted by the larger numbers in white. There are five significant correlations from the genu, four from the body, and none from the splenium.

**Fig. 6.**
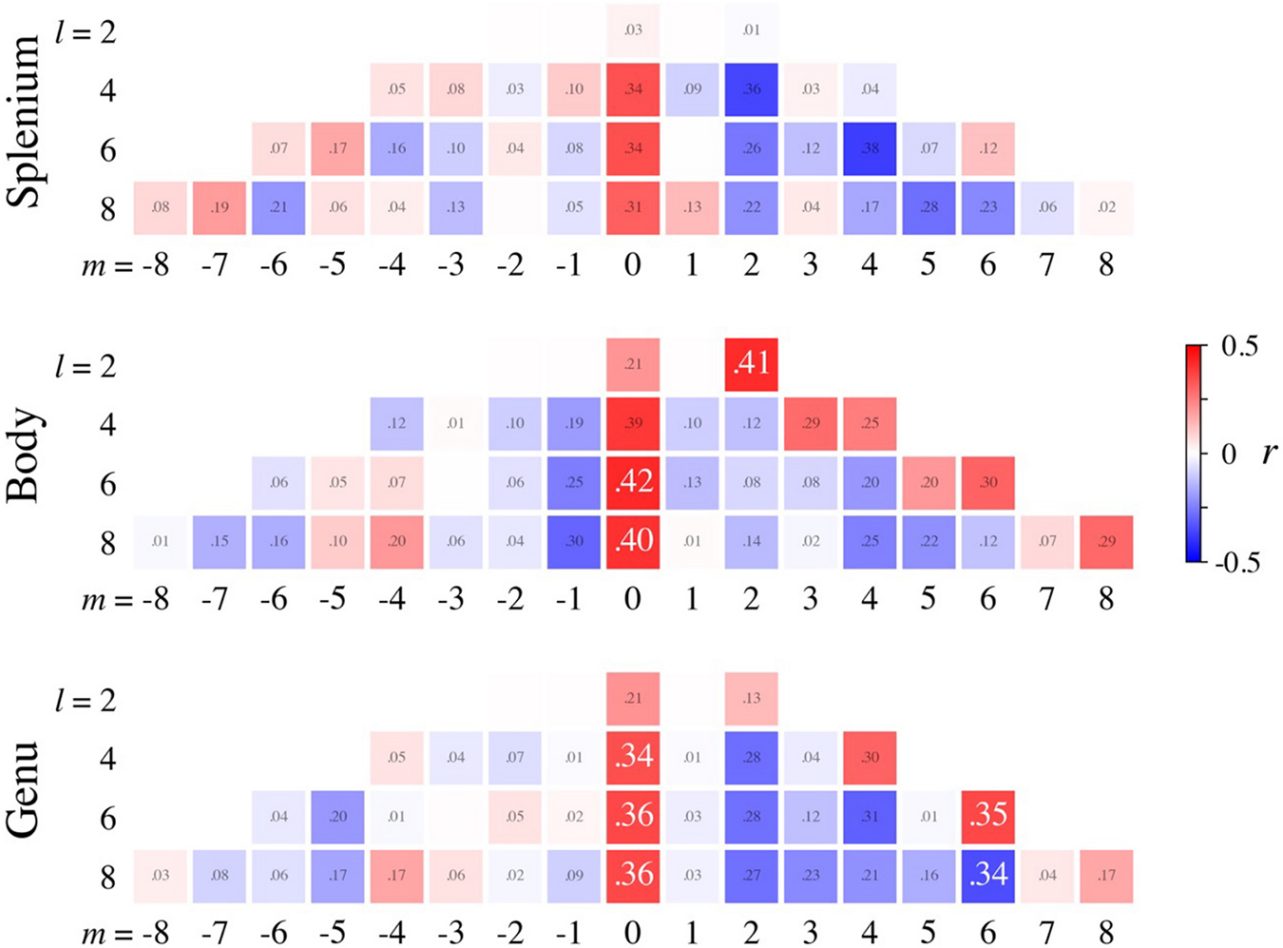
Correlation with age for the spherical harmonic expansion coefficients from the individual participants. The numbers in the squares show the magnitude of the correlations while positive correlations are in red and negative correlations in blue. The larger numbers in white are for correlations that are statistically significant after correction for multiple comparisons. Out of the non-zero coefficients for each region, five are significant in the genu, three are significant in the body, and none is significant in the splenium. This suggests that some aspects of fODF structure are more strongly associated with aging in the genu and body than in the splenium. The real form of the spherical harmonic coefficients was used for these calculations.

To extend the analysis beyond a single direction, we utilized information from all fODF directions in a simple linear regression model with age (Equation 1). The linear regression parameters for our model of the mean fODF age dependence are given inFigure 7as HEAP maps for each CC region. The first row shows the fODFs averaged over all participants$\bar{F}(\text{u})$while the second row shows the rate of change*G*(**u**). Both of these take on their largest values near the centers of the maps. The peak rate of change is similar across the regions: body (0.30 ± 0.10 %/yr), genu (0.30 ± 0.09 %/yr), and splenium (0.21 ± 0.08 %/yr) (Student’s*t*-test; splenium vs. body,*p*= 0.46; splenium vs. genu,*p*= 0.48). The Pearson correlation coefficients (bottom row) take on substantial positive values near the center that are equal to those shown inFigure 5for$F_{0}$. Large correlations are also seen in other directions, particularly close toθ=90°, but their physical relevance is limited since the fODFs have small amplitudes away from the center peaks.

**Fig. 7.**
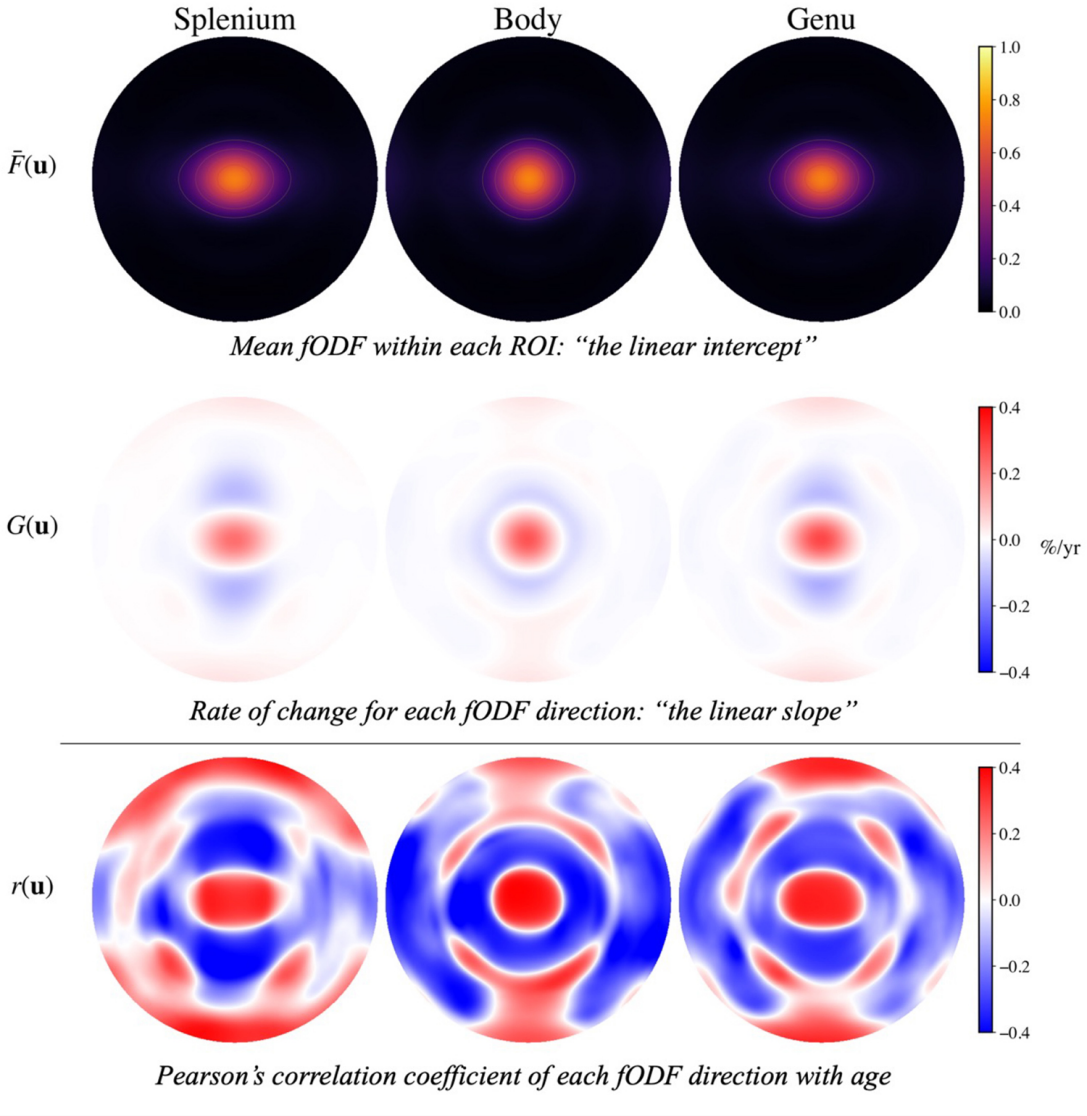
The linear regression parameters (Equation 1),$\bar{F}(\text{u})$andG(u), representing the mean fODF (top row) and the fODF rate of change with age (middle row), as functions of the directionuare displayed as HEAP maps for each CC region. The corresponding Pearson correlation coefficient for each fODF directionr(u)with age is also displayed (bottom row). The predicted rate of changeG(u)is highest near the center of the fODFs for the body and genu. The contour lines visible on the HEAP map for$\bar{F}(\text{u})$represent amplitude bands and are only for visualization.

The predicted mean fODFs calculated with our linear model are displayed inFigure 8for the ages 45, 65, and 85 years. A distinct sharpening of the peaks with increasing age is evident for all three CC ROIs. The predicted fODFs are qualitatively similar to the individual fODFs ofFigure 4but reflect the age dependence of the full group of participants. The profiles for these same predicted fODFs along both the*x*-axis and*y*-axis are plotted inFigure 9. One sees that the center peak heights grow with age in all three regions. In addition, the profiles along the*x*-axis show subtle decreases forθ≈±30°that also contribute to the peak sharpening. Along the*y*-axis, the decrease in the FWHM appears to be mainly a consequence of the increase in maximum amplitude with little change in the fODF amplitudes near 30°. It should be emphasized that all fODFs are normalized to unit integral over all directions so that an increase in peak height reflects a greater probability of axons being orientated at angles nearθ=0°but not necessarily that there are more axons aligned in this direction (since the total number of axons may decrease with age).

**Fig. 8.**
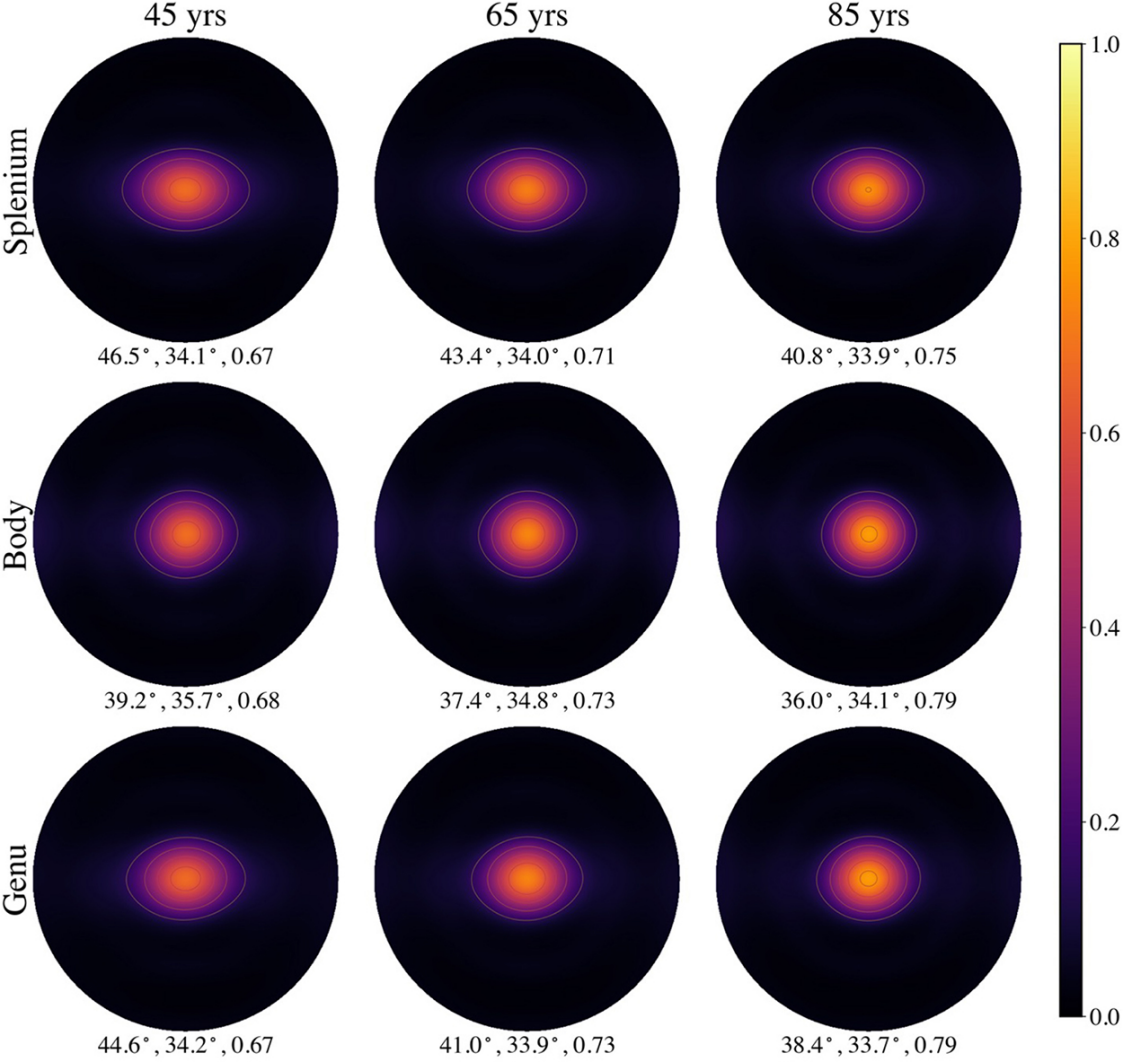
HEAP maps of predicted fODFs in three CC regions at ages of 45, 65, and 85 years. As age increases, a sharpening of the center peak is apparent in all three regions. These maps differ from those inFigure 4in being predictions based on linear regression of the full study cohort rather than measured fODFs for individual participants. The three numbers listed below each HEAP map indicate FWHM*_x_*, FWHM*_y_*, and$F_{0}$. The visible contour lines on the HEAP maps indicate amplitude bands and are only for visualization purposes.

**Fig. 9.**
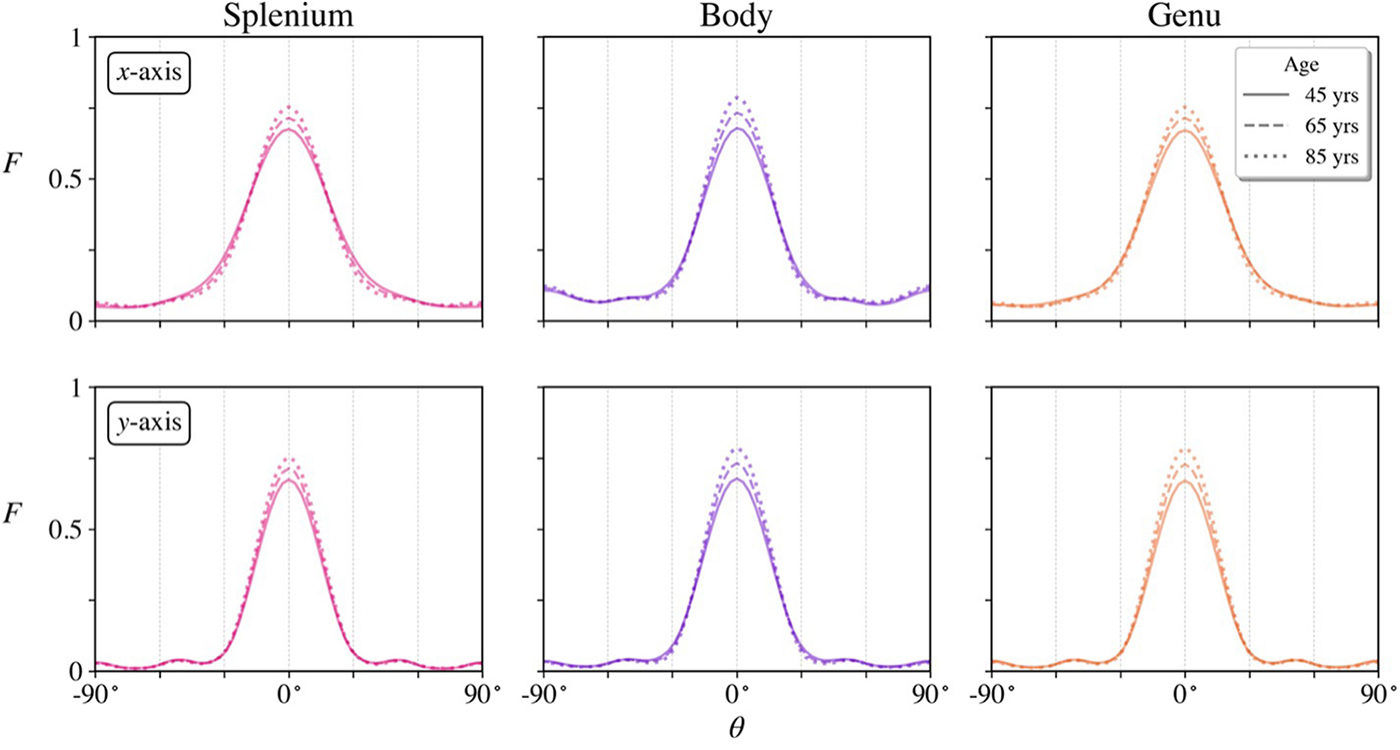
Cross sections of predicted fODFs along*x*-axis and*y*-axis for ages 45, 65, and 85 years in three CC regions. The center peak heights increase with age while atθ=±30°, the fODFs change with age along the*x*-axis more than for the*y*-axis.

The predicted dependencies of FWHM*_x_*and FWHM*_y_*with age are given inFigure 10. Along the*x*-axis, the angular dispersion decreases between ages 45 and 85 years by 5.7°, 4.2°, and 6.4° for the splenium, body, and genu, respectively. Along the*y*-axis, age-related changes are negligible, being only 0.3°, 1.4°, and 0.8°. The dashed lines indicate the angular resolution for our method of fODF estimation, which might affect these results. The predicted dependencies of the peak heights are not shown in this figure since these are identical to the best-fit lines for$F_{0}$inFigure 5. In contrast, the prediction curves for FWHM_*x*_and FWHM_*y*_are not identical to the best-fit lines inFigure 5due to their nonlinear dependence on the fODFs.

**Fig. 10.**
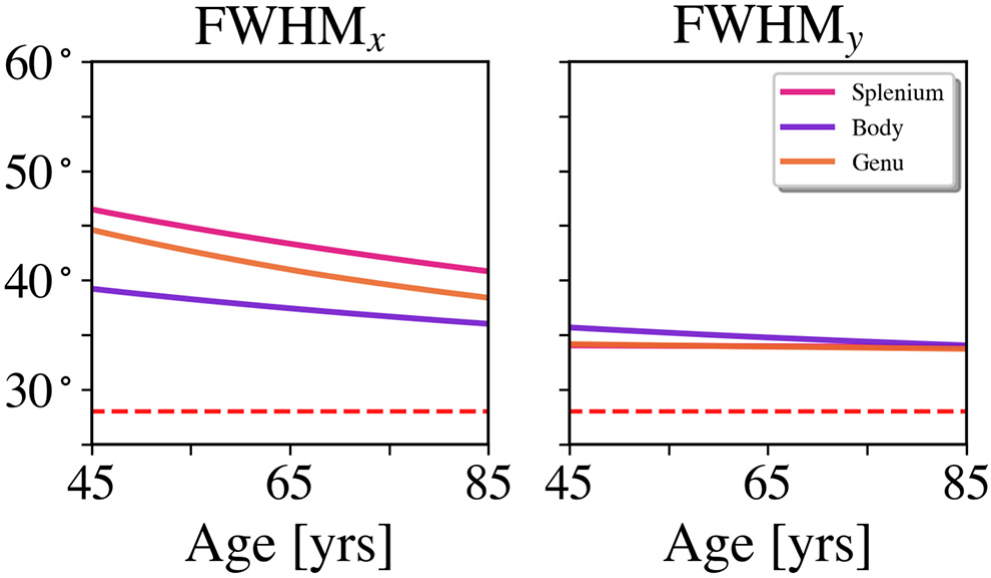
Predictions for FWHM*_x_*and FWHM*_y_*as a function of age for all three ROIs. Between ages 45 and 85 years, the angular dispersion is predicted to decrease along the*x*-axis by 5.7°, 4.2°, and 6.4° for the splenium, body, and genu, respectively. Along the*y*-axis, the predicted decreases are only 0.3°, 1.4°, and 0.8°. In the right panel, the lines for the splenium and genu overlap making them hard to distinguish. The dashed lines indicate the angular resolution for our analysis.

## 4 Discussion

Our results show that the intra-voxel angular dispersion of axonal fibers within the CC tends to decrease with age in healthy adults who are between 45 and 85 years old. The angular dispersion was quantified separately inside the splenium, body, and genu by using the FWHM of the fODF as estimated from FBI. Since fODFs typically are not axially symmetric, the FWHM was calculated along the*x*-axis and*y*-axis in the local frame of reference. For all three regions, a statistically significant decrease occurs along the*x*-axis (Fig. 5). A significant decrease is also seen along the*y*-axis in the body, but there are no significant changes for the splenium and genu in this direction. In addition, fODF peak height is found to increase with age in all regions.

Linear regression was applied to the experimental data to construct a model for predicting the fODF at any age that can serve as a normative reference in future studies of health or disease. According to this model, the FWHM along the*x*-axis is expected to decrease 4° to 7° between 45 years and 85 years of age, while the expected reduction along the*y*-axis is less than 2°. However, these results may be biased by the finite angular resolution of the estimated fODFs, which depends on the number of terms included in the spherical harmonic representation. In our analysis, we kept all terms up to and including degree 8, corresponding to an angular resolution of about 28° (Moss & Jensen, 2022a). Because higher angular frequency components are neglected, features of an estimated fODF may be smoother and broader than for an exact fODF. Hence, the actual reduction in angular dispersion with age could be larger than our measurements indicate. This is particularly true along the*y*-axis where the predicted FWHM is only about 5° to 7° larger than the angular resolution. Nonetheless, the qualitative trends found from our data should hold independently of this limitation but would likely be more difficult to discern from lower fidelity fODF measurements.

Overall, our observed age-related fODF changes are qualitatively similar for the splenium, body, and genu. Nevertheless, the rate of change in the splenium is noticeably lower, as can be appreciated inFigure 7. Moreover, the correlations for the spherical harmonic expansion coefficients (Fig. 6) reveal several significant associations with age in the body and genu but none in the splenium, again suggesting regional differences in the effects of aging on fODF structure. Such differences might be related to the known variability of axon diameter and degree of myelination within the CC (Aboitiz et al., 1992,^1996^;Lamantia & Rakic, 1990). For example, the genu has relatively more small diameter axons and is less heavily myelinated, which could make it more vulnerable to aging effects. This is consistent with our data, which show that the genu has the most significant associations with age.

Our estimated peak widths may be compared with the histological measurements ofRonen et al. (2014), who found the standard deviation (σ) of the fODF to be 18.1° in the CC. Assuming a Gaussian distribution of fiber orientations, we have FWHM =$2\sigma \sqrt{2\ln2}$= 42.6°. As an approximation based on the conventional error propagation formula used in statistics, the FWHM for an estimated fODF should be roughly the root sum square of the angular resolution and exact FWHM. The histological value then implies an FWHM of about 51° for an estimated fODF obtained at an angular resolution of 28°. This is similar to our results along the*x*-axis, where the predicted FWHM varies between 36° and 47° depending on age and location.

The age-related decrease in angular dispersion of the axonal fibers resembles the histologically observed increase in average diameter of myelinated axons with age that results from preferential loss of thinner myelinated axons (Marner et al., 2003). While our data are insufficient to identify an exact cause for the reduced angular dispersion of the CC axonal fibers, inFigure 11, we provide visual depictions of what we believe are two plausible scenarios. Indeed, the reduction in angular dispersion could itself be a direct consequence of the loss of thin myelinated axons, as would be the case if thicker axons within the CC were more directionally coherent than thinner ones. This scenario is depicted inFigure 11(Scenario A) showing how the loss with aging of thinner, less directionally coherent myelinated axons leads to a narrower overall angular distribution. The loss of myelinated axons could arise from either demyelination or axonal loss. While not definitively established, fODFs measured with FBI are believed to reflect primarily myelinated axons, presumably because the intercellular water exchange rate for unmyelinated axons is too fast to effectively compartmentalize water during the dMRI signal acquisition (Moss et al., 2019). An alternative possibility, also shown inFigure 11(Scenario B), is that thin myelinated axons with directions deviating further from the predominant direction of the fiber bundle are preferentially lost in aging compared with thin myelinated axons with smaller deviations. However, we consider this to be a less likely scenario in the absence of a plausible biological mechanism for differential axonal degeneration depending on relative orientation. AlthoughFigure 11is an idealization of a highly complex process, as a visual aid, it helps give an appreciation for the significance of the study by clarifying two hypotheses that may guide future investigations.

**Fig. 11.**
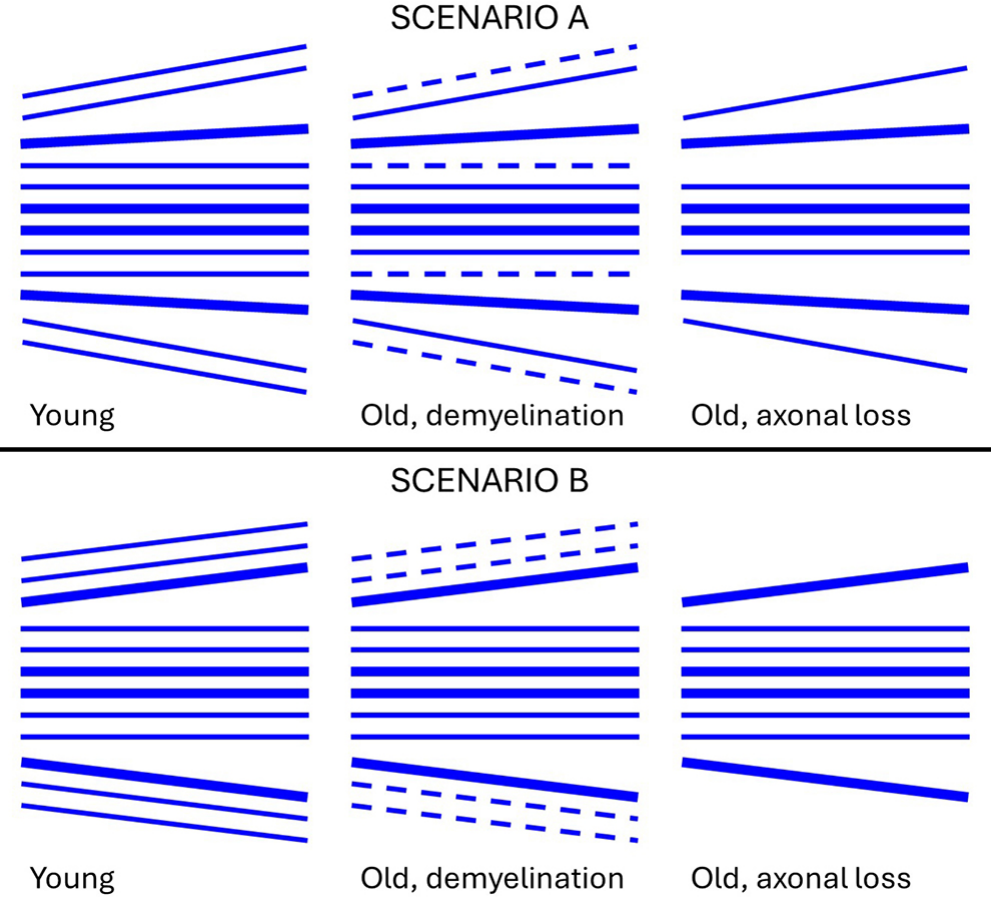
The number of thin myelinated axons (thin solid blue lines) in white matter having diameters less than about 1.5 μm decreases with healthy aging, while the number of myelinated axons of larger caliber (thick solid blue lines) is mostly preserved (Marner et al., 2003). The loss of myelinated axons can be due to both demyelination and axonal loss. In Scenario A (first row), thick axons are assumed to be more directionally coherent than either thin myelinated axons or thin unmyelinated axons (dashed blue lines). The loss in aging of thin myelinated axons either by demyelination or axonal loss then results in a narrower distribution of orientations for the remaining myelinated axons, which reduces the fODF angular dispersion. In Scenario B, thin and thick myelinated axons are assumed to have similar angular dispersions at young ages, but thin myelinated axons with orientations deviating further from the predominant direction of the fiber bundle are preferentially lost for older ages. This also implies a reduction in fODF dispersion, but presupposes a mechanism for enhanced axonal degeneration tied to axon orientation which is unnecessary in Scenario A.

In principle, three-dimensional histology based on electron microscopy could help resolve this indeterminacy by using methods similar to those of prior studies of axon geometry in white matter (Lee et al., 2019,^2024^). A study byMollink et al. (2017)further corroborated this finding within the midline CC using fODFs derived from dMRI, polarized light imaging, and histology. In particular, they identified a distinctive low-high-low dispersion pattern from the lateral to medial aspects of the midline CC. We find a similar dispersion profile as Mollink et al. in the voxel-wise FWHM_*x*_within the CC (Fig. 3). Without a histological reference for comparison, this helps substantiate our present findings even at our coarse spatial resolution.

Our observation of decreased angular dispersion is related to previously published results for age-related changes in fractional anisotropy axonal (FAA) that were obtained from the same dataset (Dhiman et al., 2022). The FAA is conceptually similar to the well-known FA, which is often measured with DTI or DKI, except that FAA only reflects diffusion anisotropy of water within axons while FA is also affected by extra-axonal water (McKinnon et al., 2018;Moss et al., 2019). Within the splenium, FAA was seen to increase significantly in healthy aging, which is in qualitative agreement with the decrease in axonal angular dispersion found here, but no significant change in FAA was found for the genu. An essential difference between FAA and angular dispersion, as quantified by the FWHM of the fODF, is that FAA depends only on spherical harmonic coefficients up to degree 2, whereas angular dispersion reflects contributions from all degrees included in the analysis (up to degree 8 in this study). Therefore, angular dispersion is more sensitive to fine structural details that are encoded by the higher degree terms of an fODF’s spherical harmonic expansion.

The greater sensitivity provided by considering all available spherical harmonic coefficients for an fODF could be advantageous for investigating subtle microstructural changes in white matter associated with neuropathologies. For instance, one ex vivo study found a substantial decrease in the percentage of axons with diameters greater than 2.2 μm within the anterior portion of the CC for patients with Alzheimer’s disease in comparison with control subjects (Køster et al., 2018). Under Scenario A ofFigure 11, this implies mean fODFs in this region should have larger intra-voxel angular dispersion for Alzheimer’s disease patients than for controls.

That diffusion measures derived from the fODF, such as the FAA and FWHM, are specific to the intra-axonal compartment enhances their biophysical interpretability. In addition, they can also help with the interpretation of more familiar quantities obtained using either DTI or DKI, such as the FA, MD, and MK, that depend on both intra-axonal and extra-axonal water diffusion. One notable example is the established age-related decrease of FA in the CC (Benitez et al., 2018;Billiet et al., 2015;Dhiman et al., 2022;Gong et al., 2014;Kochunov et al., 2012;Ota et al., 2006;Pfefferbaum et al., 2000). Since fODFs in the CC become sharper with age, the FA decrease must then be driven by changes in the extra-axonal compartment (e.g., demyelination, inflammation) that reduce diffusion anisotropy.

In the present work, we have only considered fODFs within the CC for simplicity and because of the documented age-related changes in microstructure for this part of the brain. Nevertheless, the methods applied here may be readily adapted to other white matter regions although more sophisticated approaches of assessing changes in fODFs might be necessary when intersecting fiber bundles are prominent. In particular, using the FWHM as a summary statistic could be inadequate to characterize changes in fODFs with complex structure.

While other methods of estimating fODFs are available, FBI is particularly suitable for obtaining high-fidelity representations of the angular distribution of axons, where we consider a high-fidelity representation as one with an angular resolution of better than 30° (Moss & Jensen, 2022a). The constrained spherical deconvolution (CSD) approach (Tournier et al., 2007) is similar but differs in two key respects. First, it requires a response function to be estimated using experimental data obtained from high FA white matter. Second, the fODF is calculated by an iterative numerical procedure involving Tikhonov regularization. Both of these steps are absent in FBI and replaced by the inverse generalized Funk transform, which is linear and straightforward to apply in a spherical harmonic basis (Jensen, 2022;Jensen et al., 2016;Moss et al., 2019;Moss & Jensen, 2022a). While CSD and FBI generate comparable fODFs, CSD-derived fODFs tend to have unphysical negative values over a broader range of directions, potentially due to errors associated with response function estimation (Moss & Jensen, 2021a,2021b). Moreover, the use of a response function would be especially problematic in assessing fODF structure within the CC since it contains many high FA voxels that are typically used in CSD as a reference and are assumed to have negligible fiber dispersion, thereby precluding meaningful estimation of an fODF’s FWHM. The advantage of CSD is that it is compatible with data acquired at modest diffusion weightings (*b*< 4000 s/mm^2^), while FBI is only valid for strong diffusion weightings (*b*≥ 4000 s/mm^2^), which can be challenging to implement on some scanners. However, if high-fidelity fODFs are desired, strong diffusion weightings should, in any case, be used whenever feasible to reduce the noise variance of the higher degree spherical harmonic coefficients (Moss & Jensen, 2022a).

Alternatively, fODFs can be derived from some compartment models, but this typically involves restrictive a priori assumptions about the functional form of the fODF and the extra-axonal space and may also involve nonlinear numerical fitting (Anderson, 2005;Assaf & Basser, 2005;Jespersen et al., 2010). For instance, NODDI assumes a Watson distribution for the fODF (Zhang et al., 2012), while the ball-and-racket model (Sotiropoulos et al., 2012) assumes a Bingham distribution. Moreover, both require initial estimates and fixing of the compartmental diffusivities before performing nonlinear fits from which to quantify fiber dispersion using the ODI; NODDI results in studies of aging are found to be directionally inconsistent within the CC (Billiet et al., 2015;Motovylyak et al., 2022;Raghavan et al., 2021) and the SNR requirements of the ball-and-racket model (SNR ≥30) make it unsuitable for in vivo clinical application.

As previously mentioned, estimated fODFs obtained in our analysis have an angular resolution of 28°, which may obscure some fine structural details and alter the observed peak widths. The achievable angular resolution with FBI is constrained by the number of diffusion directions sampled in the data acquisition (Moss & Jensen, 2022a). For this study, we used 128 directions, which implies a lower bound of 16.4°. However, oversampling by a factor of 2 to 3 is necessary to avoid aliasing artifacts (Moss & Jensen, 2022a,2022b). Here, our angular resolution was fixed by choosing an oversampling factor of 2.8. Similar resolution constraints apply to other fODF methods based on a spherical harmonic expansion and represent a basic limitation on the ability of dMRI to estimate fODFs. Attaining angular resolutions higher than the limit set by the number of diffusion directions is only possible if additional assumptions are imposed on the form of the fODF as is done, for example, in super resolution approaches (Tournier et al., 2007).

Reduced structural connectivity from age-related axonal degeneration, as observed with histology, may contribute to cognitive decline, even in healthy aging. Primarily affecting small-diameter axons, neurodegeneration is particularly pronounced in late myelinating brain regions, such as the genu of the CC (Aboitiz et al., 1992;Marner et al., 2003). In contrast to histology, dMRI enables the in vivo assessment of structural connectivity using the fODF, which reflects axonal organization. With high-fidelity fODFs, subtle changes are observable in the fine details of white matter microstructure because, unlike other axon-specific quantities like the FAA, the fODF contains high-frequency angular information. Our results in the midline CC show that changes occur to the fODF during healthy aging, although this has yet to be verified in other white matter regions. Nevertheless, our results are qualitatively similar to prior post-mortem studies of dispersion (Mollink et al., 2017) and could serve as a normative reference in studies of age-related neurological disorders, such as Alzheimer’s disease. With improving scanner technology, the high*b*-value dMRI data required to estimate high-fidelity fODFs using FBI should become increasingly practical to obtain, allowing the fODF to be applied in the assessment of aging and white matter disease.

A notable strength of this study is that all participants were carefully screened to minimize the occurrence of incipient or asymptomatic neurological disease that might cause brain changes unrelated to healthy aging. A limitation is the large voxel size (3 mm isotropic), which may introduce partial volume effects; however, FBI employs only large*b*-value dMRI data in estimating the fODF which strongly suppresses any signal contributions from CSF and GM. A weakness is that the extent to which the fODF estimated with dMRI is affected by unmyelinated axons is uncertain. However, the view that unmyelinated axons do not contribute substantially to the fODF is supported by the scaling with*b*-value of the dMRI signal in gray matter (McKinnon et al., 2017;Olesen et al., 2022;Veraart et al., 2018), where unmyelinated axons are abundant (Chklovskii et al., 2002). Specifically, the fODF in white matter would be expected to primarily reflect myelinated axons if the intercellular water exchange time for unmyelinated axons was small compared with the echo time for the dMRI sequence (95 ms in this experiment), as the gray matter scaling behavior suggests may well be the case.

## 5 Conclusion

The intra-voxel angular dispersion of axonal fibers within the CC decreases with age in healthy older adults between 45 and 85 years old. The known loss of thinner myelinated axons with aging could account for the reduced dispersion, as long as thicker axons are more directionally coherent. Our experiment demonstrates how subtle differences in the microstructural organization of axons across participants can be detected with dMRI by using strong diffusion weightings to estimate high-fidelity fODFs.

## Data Availability

The data employed in this study are not publicly available. The code for fODF linear regression is available upon request. The PyDesigner software used for preprocessing and image analysis may be downloaded from:https://pydesigner.readthedocs.io/en/latest/index.html
